# General practice – a fertile lagoon in the ocean of medical knowledge

**DOI:** 10.1080/02813432.2021.2004831

**Published:** 2021-11-16

**Authors:** Kirsti Malterud, Harald Kamps

**Affiliations:** a Research Unit for General Practice, NORCE Norwegian Research Centre, Bergen, Norway; b Department of Global Public Health and Primary Care, University of Bergen, Bergen, Norway; c The Research Unit and Section of General Practice, Department of Public Health, University of Copenhagen, Copenhagen, Denmark; d Lebendig Altern – Möckernkiez, Berlin, Germany

**Keywords:** (MeSH): General practice, knowledge, clinical decision-making, diagnosis, therapeutics, uncertainty

## Abstract

General practitioners (GPs) often find that linear, deductive knowledge does not provide a sufficient map for clinical management. But experience, accompanied by enduring familiarity with individual patients, may offer unique complementary skills to interpret a patient’s symptoms and navigate skilfully through diagnosis, treatment, follow-up and prevention.

In this article, we draw attention to the nature of this tacit knowing that is executed by many GPs every day. We argue that the nonlinear, unpredictable complexity of this domain nurtures a particular logic of clinical knowing. This kind of knowledge is not intuition and can to some extent be intersubjectively accessible. We substantiate and discuss how and why general practice research can contribute to knowledge development by transforming reflection-in-action to reflection-on-action.

We briefly present some concepts for reflection-on-action of clinical knowing in general practice. The VUCA model (volatility, uncertainty, complexity, ambiguity) embraces dynamic and confusing situations in which agile work (adaptive, flexible and responsive behaviour and cognitive creativity) is assumed to be an appropriate response. Using such perspectives, we may sharpen our gaze and apply reflexivity and analytic elaboration to interpret unique incidents and experiences and appreciate the complexity of general practice. In this way, exploratory research can fertilize general practice and offer innovation to the entire domain of clinical knowledge.

## Introduction

Navigating in the ocean of medical knowledge, the general practitioner (GP) is supported by knowledge from biomedicine and epidemiology – the anchorage points of evidence-based medicine. GPs find that linear, deductive knowledge does not always offer a sufficient map for sailing in the intricate waters of general practice [1,2]. However, experience can combine with enduring familiarity with individual patients to add to the skills needed to interpret a patient’s symptoms and navigate skilfully through the shoals of diagnosis, treatment, follow-up and prevention [3–6].

## GPs recognize substantial anchorage points for knowing

*Epistemology* is the science of knowledge. We argue that general practice is a clinical discipline where GPs’ proficiency represents substantial knowledge, appropriate for sailing on seas where charts are missing and decisions are made under uncertainty [7,8]. We compare knowing in general practice with knowing elsewhere in clinical practice and highlight differences and similarities. We emphasize that the clinical epistemology developed by GPs requires reflexivity and analytic elaboration to fertilize general practice and offer innovation to the entire domain of clinical knowing [2].

Applying a ‘lagoon’ metaphor, we indicate how GPs recognize anchorage points of knowing behind the perilous reefs of malpractice and casual routes of intuition [1,9]. Through skilful and reflexive navigation, GPs can perceive, challenge and share such clinical knowing. This lagoon is not a convoluted, divine domain. Paradigms for interpretation and understanding human subjectivity are needed to complement ideas of universal objectivity [2,3]. We thus envision a palpable beach with smooth sand and irregular pebbles, ready for the GP to explore.

## Assignments and framework specific for general practice

Let us compare this lagoon to the coastline of the medical ocean. By seeing patients over time in their everyday practice rather than casual hospital encounters, GPs establish a unique personhood for the contextual interpretation and situated knowing of each patient [4,10]. The emergency doctor must take action immediately, while the GP – accustomed to a variety of symptoms and diseases – is trained to sort illness and disease with the aid of time [6]. The ophthalmologist usually responds to a single diagnosis, while multimorbidity is common among the GP’s patients. The oncologist must impart pathological findings and deliver painful news, while the GP often concludes that a given problem is not serious or will soon disappear. The radiologist needs advanced technology, while the patient’s story can be the most important diagnostic tool to the GP. These features of general practice also appear, although inconsistently, in paediatrics, geriatrics and psychiatry.

Furthermore, general practice is part of but not identical to primary care. GPs share community knowledge by collaborating with nurses, social workers, physiotherapists, psychologists and welfare officers. Specific assignments for GPs include diagnosis, treatment, follow-up and prevention of unsorted diseases, both common and rare, presented within a doctor-patient-relationship that includes the patient’s family and everyday life [4]. These advanced tasks require extensive medical training and experience [5,6]. In general practice, evidence from research findings is adapted and individualized. The GP supports public health strategies, but prevention and health promotion with the particular patient always put values at stake, sometimes overriding evidence [10,11]. In general practice, patients frequently present unexplained or intermediate bodily symptoms between normality and pathology [1]. Among the elderly, polypharmacy is the rule. While the complexity of this medical seascape offers many challenges, it offers the proficient GP the benefits of confidence and variety [12–14].

## The fertile lagoon of general practice – a laboratory for knowing and reflexivity

*Ontology* is the science of being. Because general practice is embedded in everyday life, this medical speciality represents a specific ontology with the power to challenge and transform medical knowing and epistemology [3]. Navigating unpredictable currents demands a steady (but not static) course, a pragmatic (though well-read) competence and a confident (yet not obstinate) capacity for uncertainty to complement the facts. Through experience and interaction with patients and colleagues, the GP cultivates a gaze for unexpected cues, indicating when a diagnostic path is a dead end, while other subliminal cues hint at unique interpretations of signs and symptoms [15–17]. The fertile lagoon of family practice offers a laboratory for recognizing, elaborating and charting such skills, although they are not immediately observable among the pebbles on the beach. Without the appropriate gaze and language, this ontology with these treasures can hardly be explored, delineated and described. They remain hidden behind a reef that nobody can locate. So how can we grasp and discipline the subjectivity required for the interpretation of such uniqueness?

Encountering the dynamic challenges of clinical decision making, epistemology is often confronted and expanded as *tacit knowing* [18]. Clinical rationality has been described as *phronesis*, or practical reasoning in the Aristotelian sense [1]. This epistemological backyard is often referred to as intuition, ‘gut feelings’ or the art of medicine, implying a magical subjectivity outside the respectable company of academic medicine [2,9]. The GP’s relationship with the patient is the foundation of the medical context and ontology [4], but the subjectivity situating GPs as human beings and supporting interpretations and behaviour is far from casual.

This nonlinear, unpredictable context of knowing can become intersubjectively accessible and thus compatible with its complexity [13]. The key to an appropriate epistemology is *reflexivity*, situating the knowledge according to the perspective of the knower and considering the knower’s participation in its creation [14,17]. Schön describes practitioners exercising reflection-in-action as unarticulated conversations with the situation to translate complex problems into solvable formats [19]. By articulating and transforming reflection-in-action to *reflection-on-action*, experiences can be shared, discussed and elaborated.

## Volatility – uncertainty – complexity – ambiguity (VUCA)

Let us propose some concepts to sharpen the gaze for reflection-on-action. The *VUCA model* (volatility, uncertainty, complexity, ambiguity) was coined by US military leaders to describe strategic challenges in the less clear-cut situation that followed the fall of the Soviet Union. VUCA has since been adopted by the corporate world in a leadership and management context [20]. *Agile work*, a concept from software development to describe adaptive, flexible and responsive behaviour and cognitive creativity [21], has been suggested as a response to VUCA challenges [20]. These concepts can support the exploration of the lagoon of general practice.

On a sunny day, the GP glides with a tailwind where symptoms match therapeutic options. On another day, information sources are like volatile winds blowing from changing directions, much as everyday practice is transformed from day to day by the COVID-19 virus. Perhaps the GP must tack against the wind in approaching rough waters where the patient’s headache does not fit into any known map and causes uncertainty. The next day, the sea is more churned. We have no horizon and fear losing our direction due to complex currents. It is difficult to decide whether the patient with the derailed blood glucose, the one facing unemployment or the one with the failed relationship calls most urgently. Finally, the sunset reflects the ambiguity of a familiar landmark, leading to faulty navigation, as when our official guidelines are pointing in mutually opposite directions, or when the GP mistakes an intestinal tumour for trivial constipation.

## Encountering complexity with agile working

These stories may appear frightening. To the GP, however, agile work and reflection-in-action are immanent everyday skills rather than fears to be avoided [15]. GPs admit that navigational tools can offer conflicting information. All laboratory tests may be normal, but we still ‘know’ that something is wrong. We notice that the patient is depressed and suffering, but he insists that everything is fine. Indeed, the GP sometimes does not need to feel safe at all. Symptoms are allowed to remain vague, and the GP trusts that time will soon tell. Repeated experiences of uncertainty teach us to navigate as safely as possible, continuously considering whether we should be alarmed and change course or whether we can await progress in the right direction [8]. According to Rudebeck, "...the challenge lies in understanding and identifying that general clinical competence which mediates between the individual patient and biomedicine and which contributes to the competence of the skilful clinician irrespective of specialisation" [22].

One morning in the office will provide ample examples of the volatile, uncertain, complex and ambiguous work that makes up general practice. These examples demonstrate how general practice can be an exciting journey instead of a perilous route [12]. VUCA can become the symbol of a challenging and rewarding medical discipline in which agile working is an essential skill. Navigating the ocean of medical knowledge towards the lagoon of general practice can be daunting for newcomers unless they are guided by an experienced captain. For the senior GP, sailing offers opportunities to explore the lagoon more deeply, develop reflection-on-action and confidently convey general practice as a domain for creative medical knowing [2,17]. 

## Implications for practice and research

Developing the capacity for agile working has the potential to make the GP a proficient pilot for the exploration of a broad range of questions and answers [6,19]. These pursuits represent anchorage points for academic reflexivity – systematic strategies to make clinical knowing perceivable, interpretable and sharable. Still, we must not fall into scientism – a naïve belief that science can answer any question. The GP should read manuals and maps before the journey and must also improvise to change course for a specific situation. The supreme discipline of sailing is to reflect critically upon the chosen course and respond in due time if the direction no longer coincides with the goal. Through agile working in general practice, the sailor shows respect for the sea, not by ruling the waves but by mastering the craft through constant dialogue with the passengers and crew, the water and its currents, the winds and the qualities of the ship.
